# Propofol suppresses the growth and invasion of cervical carcinoma cells by inhibiting MIR155HG

**DOI:** 10.18632/aging.203697

**Published:** 2021-11-13

**Authors:** Xin-Tan Du, Xiao-Yan Wang, Ying-He Zheng, Da-Peng Liu

**Affiliations:** 1 Department of Anesthesia and Perioperative Medicine, Zaozhuang Municipal Hospital, Zaozhuang, Shandong, People’s Republic of China; 2 Department of Gynecology and Obstetrics, Zaozhuang Hospital, Zaozhuang Mining Group, Zaozhuang, Shandong, People’s Republic of China; 3 Department of Anesthesiology, Zaozhuang Hospital, Zaozhuang Mining Group, Zaozhuang, Shandong, People’s Republic of China

**Keywords:** propofol, cervical cancer, invasion, EMT

## Abstract

Background: Cervical cancer is the most prevalent malignancy worldwide and propofol reportedly has anti-cancer efficiencies. Herein, we tried to address the potential anti-cancer effects of propofol in cervical carcinoma.

Materials and Methods: The suppression effects of propofol on the proliferation and invasion of cervical cancer cells were analyzed by Cell Counting Kit-8 (CCK-8), colony formation and Transwell invasion assay. The protein expressions of epithelial marker, E-cadherin and mesenchymal marker, N-cadherin were evaluated using western blot. The level of MIR155 host gene (MIR155HG) was determined by qRT-PCR assay. The anti-cancer impact of propofol on cervical cancer cells growth *in vivo* was determined by means of xenograft tumor model and lung metastasis model.

Results: *In vitro*, propofol inhibited the growth and colony-formation of cervical carcinoma cells. Meanwhile, propofol treatment reduced the invasive trait of cervical carcinoma cells. In addition, MIR155HG was identified to be distinctly upregulated in cervical carcinoma when compared within normal. Propofol treatment decreased the expression of MIR155HG in cervical cancer cells. Consistently, the results from *in vivo* xenograft model indicated that propofol repressed cervical cancer cells growth and decreased the expression of MIR155HG *in vivo*. Furthermore, reintroduction of MIR155HG into cervical cancer cells counteracted the inhibitory potency of propofol on the growth and aggressive phenotypes in cervical carcinoma cells.

Conclusions: Altogether, these results indicated that propofol restrained the growth and invasion of cervical cancer cells partly via regulating MIR155HG expression.

## INTRODUCTION

Cervical cancer, a form of gynecological oncology, is the leading cause of cancer-related death worldwide [1]. Despite the progress in the improvement of treatment options and targeted drugs, the overall survival for patients with cervical cancer is still unsatisfactory owing to recurrence and metastasis [2, 3]. Metastatic recurrence is a leading cause of cancer-associated death, and cancer cell migration is the initial step among the metastatic process. A growing body of evidence has identified that epithelial-mesenchymal transition (EMT) plays pivotal parts in cancer cell metastatic dissemination via endowing them with a more migrate and invasive trait [4]. Hence, targeting EMT process may have important therapeutic implications in cancer management [5]. An extensive body of studies has reported that long noncoding RNAs (lncRNAs) are involved in tumor invasion and metastasis by regulating EMT [6]. Similar to protein-coding regulators, recently mounting studies have revealed that lncRNAs are also importantly involved in tumor progression and have great potentials to be clinically applied in the treatment of cancer [7].

Previous studies have revealed that some anesthetics inhibit cancer metastases, not only via blocking perioperative stress responses, but also through inducing anticancer effects [8, 9]. Prior studies have suggested that propofol (2, 6-diisopropylphenol), one of the most extensively used intravenous anesthetic agents during cancer surgical excision, influences the biological behaviors of cancer cell, including growth, invasiveness, and metastasis [10, 11]. For example, propofol restrains pancreatic cancer cells growth and metastases via elevating miR-328 and depressing ADAM Metallopeptidase Domain 8 (ADAM8) [12]. In colorectal cancer, propofol inhibits cancer cells proliferation and metastasis by regulating miR-124-3p.1/AKT3 [13]. Furthermore, propofol inhibits the proliferation and cisplatin resistance in ovarian carcinoma cell via modulating miRNA-374a/forkhead box O1 signaling [14]. However, to our knowledge there have been no further deeply investigations on the mechanisms underlying propofol in anti-cancer in cervical cancer. As propofol is a common sedative and anesthetic, it is important to understand more mechanisms about the inhibition of cancer metastasis for its usage in surgery of invasive cancer.

Herein, we confirmed that propofol restrains the growth and invasion capacity of cervical carcinoma cells. MIR155HG is remarkedly upregulated in cervical cancer and MIR155HG silencing significantly represses the progression of cervical carcinoma cells. Upon further investigations, we demonstrated that propofol restrains the growth and invasiveness of cervical cancer cells in a MIR155HG dependent manner.

## MATERIALS AND METHODS

### Cell and reagent

Caski and SiHa cervical cancer cell lines were purchased from the Chinese Academy of Sciences (Shanghai, China). Cells were maintained in DMEM (Thermo Fisher Scientific, Waltham, MA, USA) supplemented with 10% FBS and 1% penicillin/streptomycin. Cell lines were cultured in 5% CO_2_ at 37° C. Propofol (purity≥98%) was purchased from Sigma-Aldrich (Shanghai, China).

### Cell transfections

To knockdown MIR155HG, shRNA targets MIR155HG (sh-MIR155HG) was generated by GenePharma (Shanghai, China). Cells were transfected with sh-MIR155HG or shRNA negative control (sh-NC) by use of the Lipofectamine RNAiMAX Reagent (Thermo Fisher Scientific). The full length of MIR155HG was subcloned into pCDNA3.1 plasmid (Thermo Fisher Scientific) vector, generating the pCDNA3.1-MIR155HG plasmid (MIR155HG-OE) for overexpression of MIR155HG in cervical cancer cell (MIR155HG-OE).

### Cell proliferation

Cells (1 × 10^3^) were seeded into 96-well plates and cultured overnight. 24 hours later, culture medium was moved, and cells were cultured in media containing propofol. After culturing for 24 hours, 48 hours, or 72 hours, 10 μl of cell counting kit-8 (CCK-8; Beyotime, Jiangsu, China) was placed into each well. After cultured for 30 min, the absorbance value (OD) was measured at 450 nm.

### EdU (5-ethynyl-2′-deoxyuridine) staining

Untreated or MIR155HG-OE cells (2 × 10^3^ cells/well) were seeded into 96-well plates and cultured in medium supplemented with or without propofol (50 μM) for 24 h. Then, 10 nM EdU was added for 12 h and then nucleus was stained with DAPI. The wells were washed with PBS, and the cells were fixed in 4% paraformaldehyde and stained according to the manufacturer's instructions.

### Colony formation

Approximately 1 × 10^3^ cells were cultured into 6 well-plates. The medium was replaced with complete media containing propofol every three days. After incubation at 37° C for two weeks, cell colonies were fixed by methanol and dyed with 1% crystal violet. The number of cell colonies was manually counted.

### Invasion assay

The upper chamber of the Transwell chamber (8.0 μm; Coring, NY, USA) were coated with Matrigel (Corning). 200 μl of cell suspension (5 × 10^3^) were plated into the upper compartment with media supplemented with propofol. 600 μl of medium supplement with 10% FBS was added to the lower chamber. After 24 hours, the invading cervical cancer cells were fixed using 4% paraformaldehyde and stained with crystal violet (1%).

### RNA extraction and quantitative real-time PCR (qRT-PCR)

Total RNA was extracted from cells using Trizol reagent (Thermo Fisher Scientific) according to the manufacturer’s instructions. First-strand cDNA was synthesized by PrimeScript reverse transcriptase (TaKaRa Bio, Dalian, China). qRT-PCR reactions were performed using Takara’s SYBR Premix Ex Taq™ II (Tli RNaseH Plus) in Applied Biosystems 7500 Fast Real-Time PCR System (Applied Biosystems). Relative expression level of MIR155HG was calculated using the 2^–∆∆Ct^ method. The primers for qRT-PCR are listed as following: MIR155HG forward, 5′-TGGAGATGGCTCTAATGGTGG-3′; reverse, 5′-TCAGTTGGAGGCAAAAACCC-3′; GAPDH forward, 5′-TGGATTTGGACGCATTGGTC-3′; reverse, 5′-TTTGCACTGGTACGTGTTGAT-3′. The GAPDH was used as an internal control.

### Immunoblotting

Cells were lysed RIPA buffer. The lysates were separated on 8% SDS-PAGE and transferred onto PVDF membrane (Millipore, Braunschweig, Germany). After blocking with 5% non-fat milk, the PVDF membrane was incubated at 4° C overnight with E-cadherin (1:1000, Abcam, Cambridge, UK), N-cadherin (1:1000, Abcam) or GAPDH (1:1000, Abcam) for overnight. After incubation with HRP-linked anti-rabbit antibody (1:10000, Bioworld, China) for 2 hours, the target bands were detected using an ECL reagent (Bio-Rad, USA).

### Tumorigenesis assay and lung metastasis model

100 μl SiHa cells (1 × 10^6^) were subcutaneously implanted into BALB/c nude mice. Tumors were measured weekly and tumor volume (mm^3^) = (length × width^2^)/2. After the mean tumor volume reached 100 mm^3^, nude mice were classified into two groups (n=6 in each group). Mice were intraperitoneally injected with vehicle (1%, v/v, DMSO in normal saline) or propofol (20 mg/kg) once a week [15]. Tumor volume was measured every week. 35 days after treatment, mice were euthanized, and the xenograft tumors were isolated, processed for western blot and immunohistochemical staining. SiHa cells were injected into the nude mice via an i.v. lateral tail vein injection (5 × 10^6^ in 100 μL PBS/mouse). Mice were intraperitoneally injected with vehicle or propofol (20 mg/kg) once a week (n=3 in each group). Six weeks after injection, the mice were euthanized. The lung tissues were fixed and subject for H&E staining. The number of superficial lung metastatic lesions was counted. Animal experiment was approved by the Ethics Committee of Zaozhuang Municipal Hospital.

### Statistical analysis

The data are calculated by GraphPad Prism 8.0 software and presented as the Mean ± Standard deviation (SD) from at least three independent experiments. Student’s t-test or one-way ANOVA followed by Tukey’s post hoc test is used to assess differences. *P*-value<0.05 is statistically significant.

## RESULTS

### Propofol suppresses the growth and invasion of cervical carcinoma cells

**Figure 1:**
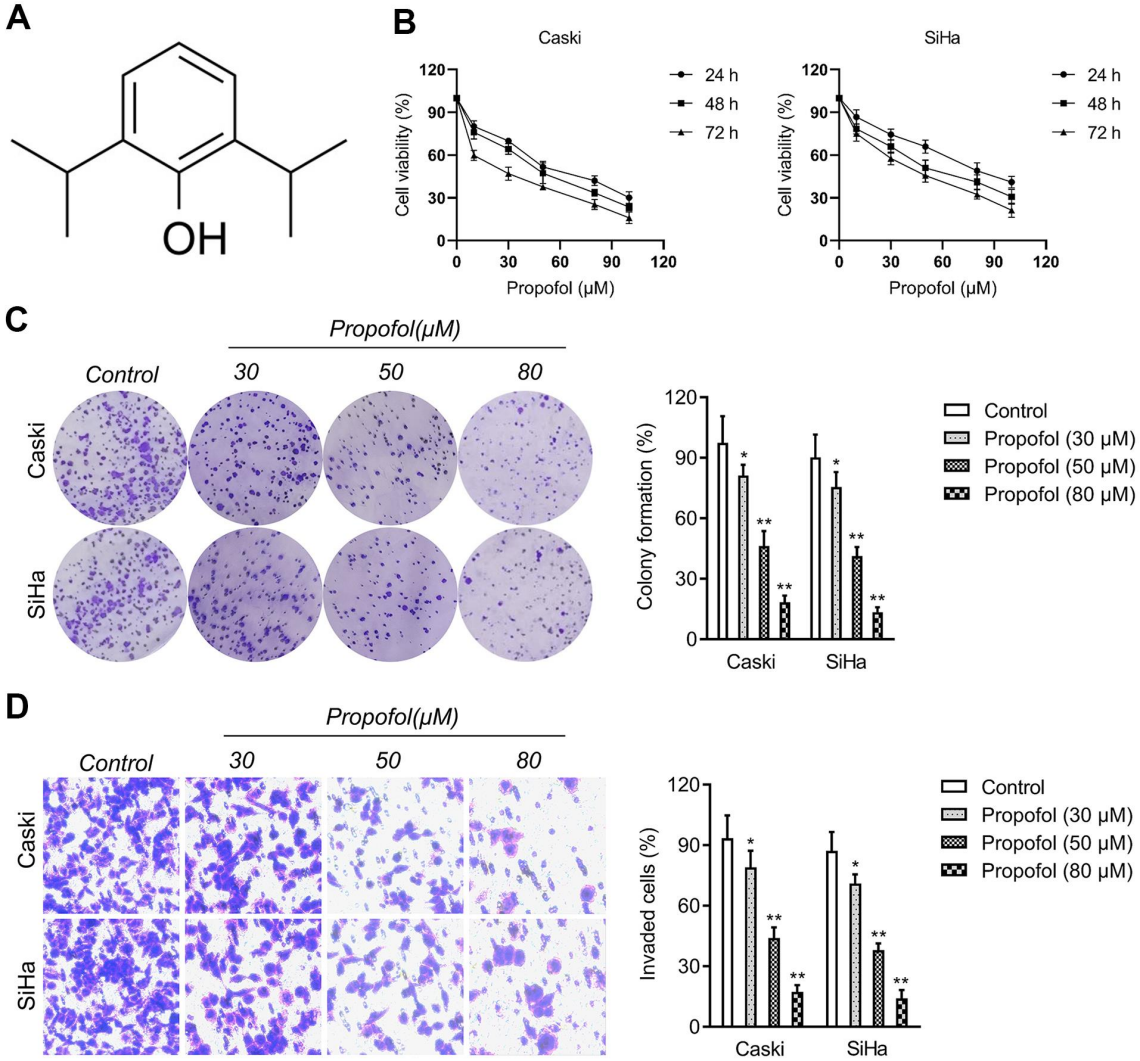
**Propofol reduces cervical cancer cell growth and invasion.** (**A**) Chemical structure of propofol. (**B**) CCK-8 proliferation assay of Caski and SiHa cells treated with different concentrations of propofol for 24 hours, 48 hours or 72 hours. (**C**) Colony formation assay of Caski and SiHa cells treated with propofol (30~80 μM). (**D**) Transwell invasion assays showed that propofol decreased the invasion ability of Caski and SiHa cells. ^*^P<0.05, ^**^P<0.01 compared with control.

Before studying the influence of propofol (Figure 1A) on cervical cancer cells, CCK-8 assays were carried out to find the suitable concentration of propofol on cervical cancer cells (Caski and SiHa). As shown in Figure 1B, propofol at 30~100 μM had the remarkedly suppressive effect on the viability of cervical carcinoma cells. Propofol at 30, 50, or 80 μM was chosen in the follow-up pharmacological evaluation. Then, we observed that propofol (30, 50, 80 μM) remarkedly reduced the colony formation of cervical carcinoma cells *in vitro* (Figure 1C). Next, we explored whether propofol altered the invasive capacities of cervical carcinoma cell. Caski and SiHa cell were treated with propofol and transwell invasion test was performed. As shown in Figure 1D, propofol reduced the invasion capacity of Caski and SiHa cell in a dose-dependent manner. These data indicated that propofol impedes the growth and invasion of cervical carcinoma cells.

### MIR155HG is upregulated in cervical cancer

**Figure 2:**
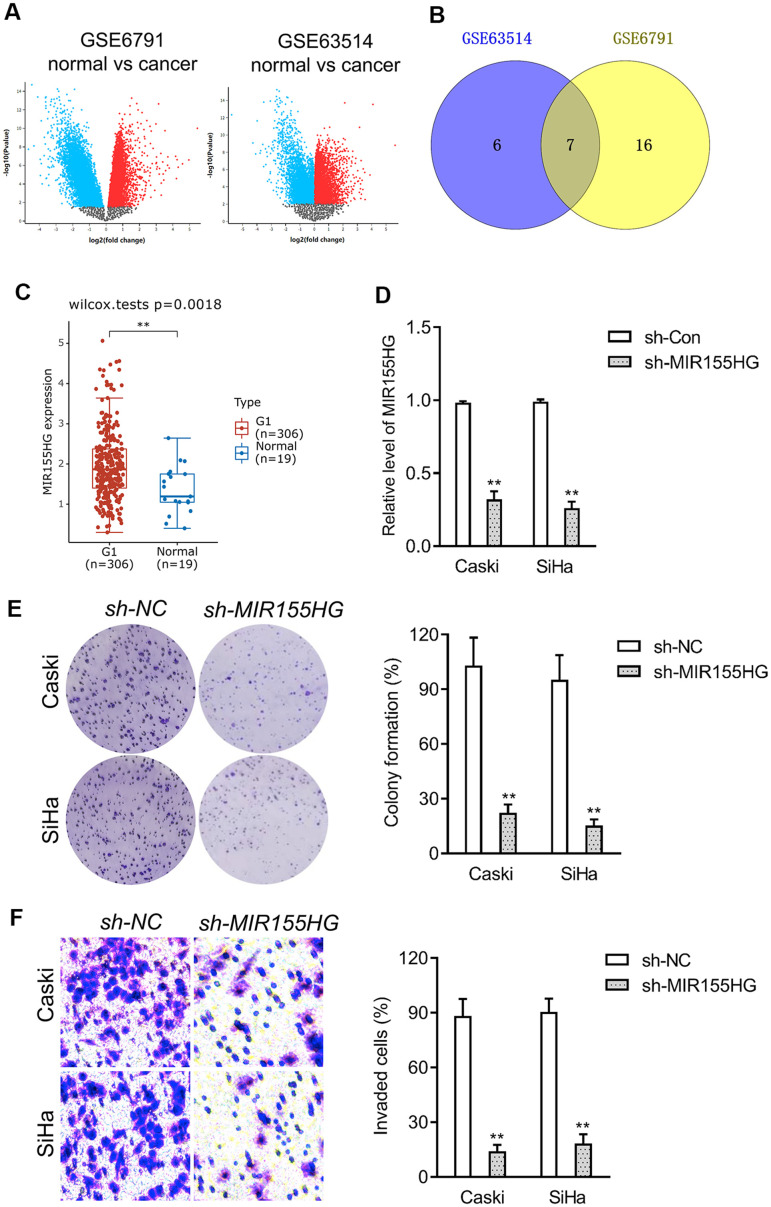
**Differentially expressed lncRNAs in cervical cancer.** (**A**) The volcano plots of GSE6791 and GSE63514 were constructed using fold-change value and P-value, and the differentially expressed lncRNAs were signed in red or blue. (**B**) Venn diagram of dysregulated lncRNAs in common from the two datasets. (**C**) The expression profiling of MIR155HG in cervical cancer tissue (G1) and normal tissue (Normal) was analyzed using GTEx. (**D**) Caski and SiHa was transfected with sh-MIR155HG. The expression of MIR155HG was determined by qRT-PCR. (**E**) The growth of cervical cancer cell was evaluated with colony formation assay. (**F**) Transwell invasion assay of MIR155HG silencing Caski and SiHa cell. ^**^P<0.01 compared with sh-NC.

To screen the potential dysregulated lncRNAs in cervical cancer, a group of differentially expressed genes was obtained from the GSE6791 and GSE63514 databases using the R package ‘limma’ (Figure 2A). 23 dysregulated lncRNAs were dysregulated in GSE6791 (|Fold change| >1 and *P*<0.05) and 13 dysregulated lncRNAs were dysregulated in GSE63514 (|Fold change| >1 and *P*<0.05). By use of the R package ‘venn’, we totally observed that 7 dysregulated lncRNAs (LINC01305, LINC00467, LINC01355, LINC00673, LINC01560, LINC01133, MIR155HG) were commonly dysregulated in these two datasets. Among these differential lncRNAs, we focused on MIR155HG due to its significance in overall survival among multiple types of cancers [16, 17]. The expression pattern of MIR155HG in cervical cancer was analyzed using Genotype-Tissue Expression (GTEx). The result revealed that MIR155HG expression was upregulated in cervical cancer when compared with in normal (Figure 2C). To investigate whether MIR155HG is associated with aggressive traits of cervical cancer cells, Caski and SiHa cells were transfected with shRNA MIR155HG (sh-MIR155HG). As shown in Figure 2D, transfection of sh-MIR155HG significantly reduced the endogenous expression of MIR155HG in both cervical cancer cells. Loss-function assay indicated that MIR155HG silencing strikingly repressed the colony formation and invasive property of cervical carcinoma cells *in vitro* (Figure 2E, 2F).

### MIR155HG expression is reduced by propofol

**Figure 3:**
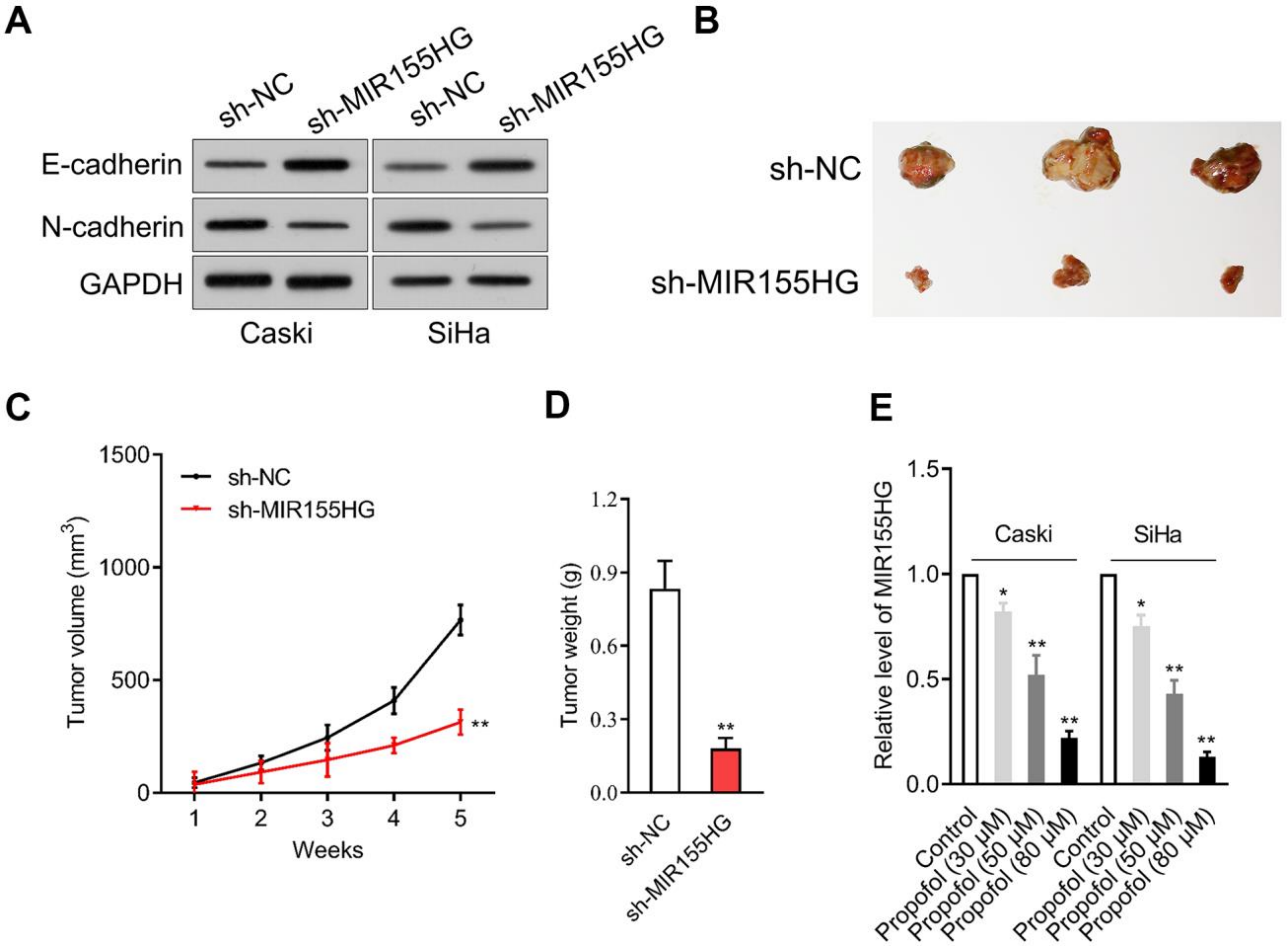
**MIR155HG expression is reduced by propofol.** (**A**) Caski and SiHa was transfected with sh-MIR155HG. The expressions of N-cadherin and E-cadherin were detected using immunoblotting. (**B**) Photographs of representative tumor tissue in tumor xenografts five weeks after subcutaneous inoculated sh-NC or sh-MIR155HG transfected SiHa cells. (**C**) Quantitative analysis of tumor volume. (**D**) Quantitative analysis of tumor weight. ^**^P<0.01 compared with sh-NC. (**E**) MIR155HG expressions in propofol-treated Caski and SiHa cells were determined by qRT-PCR. ^*^P<0.05, ^**^P<0.01 compared with control.

We further surveyed the protein expressions of E-cadherin and N-cadherin in sh-MIR155HG transfected cells by western blot assay. The experiment showed that MIR155HG silencing strikingly repressed the expression of N-cadherin and raised the expression of E-cadherin in cervical cancer cells (Figure 3A). The potential role of MIR155HG *in vivo* was tested by using a xenograft nude mice model. sh-MIR155HG transfected SiHa cells or sh-Con transfected cells were injected subcutaneously into the nude mice. We found that silencing of MIR155HG significantly inhibited SiHa xenograft tumor growth, causing significant reduction of tumor volume (Figure 3B–3D). Given the importance of MIR155HG for the invasive phenotype and EMT process of cervical carcinoma cells, we focused on the interaction between propofol and MIR155HG. To evaluate the influences of propofol on MIR155HG, qRT-PCR assay was carried out *in vitro*. As shown in Figure 3E, propofol inhibited the level of MIR155HG in cervical cancer cells in a dose-dependent manner.

### Reintroduction of MIR155HG impairs the suppressive effect of propofol in cervical carcinoma cell

**Figure 4:**
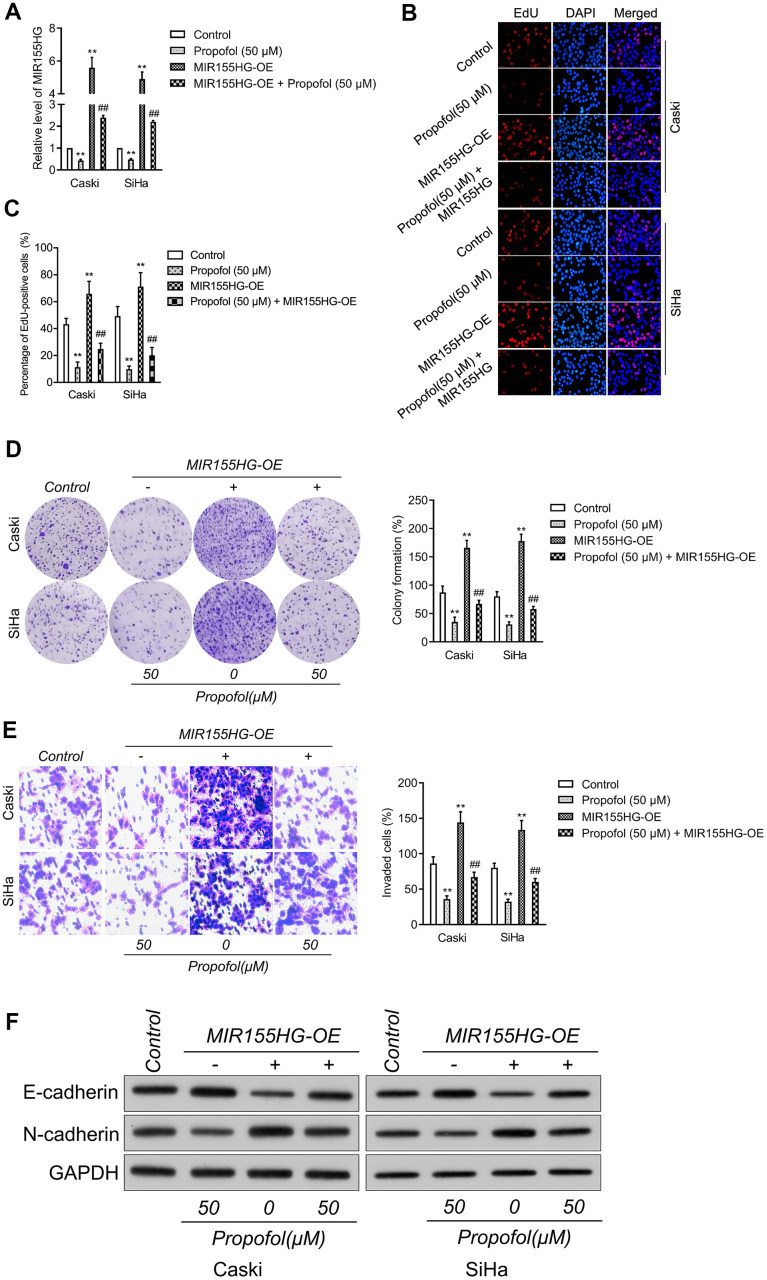
**MIR155HG mediates the anti-cancer effect of propofol in cervical cancer cell.** (**A**) MIR155HG overexpressing Caski and SiHa cells were exposed to propofol (50 μM). The level of MIR155HG was determined by qRT-PCR. (**B**, **C**) EdU staining assay showed that the proliferation of Caski and SiHa cell were increased after MIR155HG overexpression in the presence of propofol. (**D**) Colony formation assay showed that the growth of Caski and SiHa cell were increased after MIR155HG overexpression in the presence of propofol. (**E**) Transwell invasion assay showed that the invasion ability of Caski and SiHa cell were increased after MIR155HG transfection. ^**^P<0.01 compared with control, ^##^*P*<0.01 compared with propofol. (**F**) The expressions levels of EMT markers in propofol-treated Caski and SiHa cells after MIR155HG transfection as determined by western blot analysis.

To corroborate the interaction between MIR155HG, propofol and EMT, cervical cancer cell was transfected with pcDNA3.1 carrying MIR155HG (MIR155HG-OE), and then treated with propofol. The transfection was verified with qRT-PCR test (Figure 4A). Functionally, EdU and colony formation assay indicated that transfected of MIR155HG-OE rescued the growth of Caski and SiHa cell inhibited by treatment with propofol (Figure 4B–4D). Furthermore, Transwell assay demonstrated that reintroduction of MIR155HG abolished the inhibitory action of propofol on the invasive abilities of cervical carcinoma cell (Figure 4E). Furthermore, cervical cancer cells were exposed to propofol, and we found a heighten in the level of E-cadherin while a decline in the level of N-cadherin.

Nevertheless, in MIR155HG-OE group, the effect of propofol was impaired (Figure 4F). Altogether, upregulation of MIR155HG impaired the suppressive influence of propofol on the aggressiveness and EMT of cervical carcinoma cell.

### Propofol reduces the growth of cervical carcinoma cell *in vivo*

**Figure 5:**
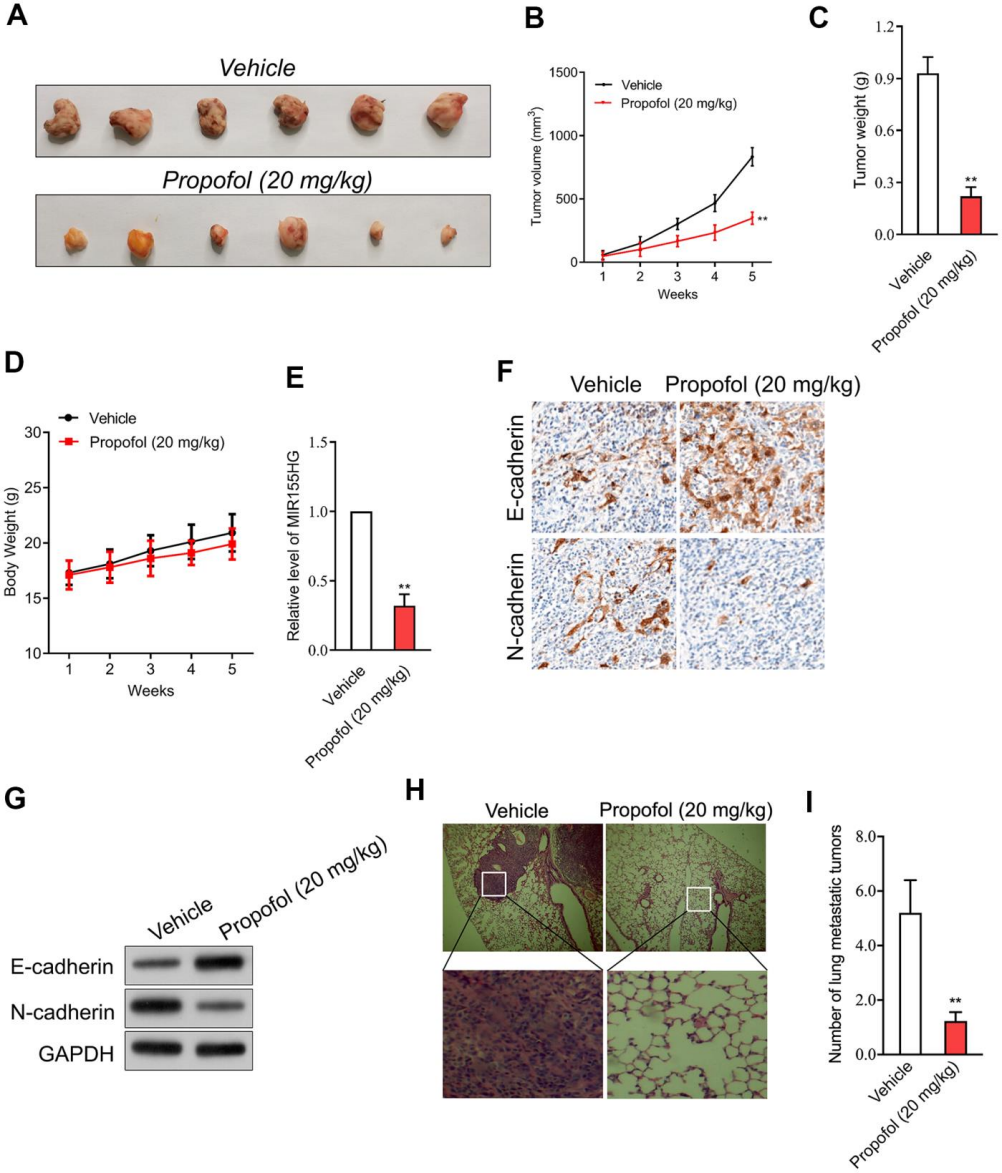
**Propofol suppresses SiHa cell growth *in vivo*.** (**A**) Photographs of representative tumor tissue in tumor xenografts five weeks after propofol treatment. (**B**) Quantitative analysis of tumor volume. (**C**) Quantitative analysis of tumor weight. (**D**) Body weight. (**E**) MIR155HG expressions in tumor tissue were determined by qRT-PCR. (**F**) The expression of E-cadherin and N-cadherin were determined using immunohistochemical staining. (**G**) Western blot analysis for E-cadherin and N-cadherin in tumor tissue. (**H**) Representative images of the histologic lung metastases were analyzed using H&E staining. (**I**) The number of lung metastatic tumors were graphed. ^**^P<0.01 compared with vehicle.

In addition, transplanted tumor model was constructed to assess the anti-cancer efficacy of propofol in cervical cancer cell growth *in vivo*. The data revealed that tumor weight and tumor growth were smaller in nude mice treated with propofol compared with that in vehicle control group (Figure 5A–5C). Meanwhile, no significant body weight loss was found in the propofol-treated mice compared to the vehicle treated group (Figure 5D). No lesions were observed on the major organs of the mice tested. These suggest that the anti-cancer effect of propofol is not dependent on its toxic side effect. Similarly, propofol also significantly reduced the level of MIR155HG in tumor tissue (Figure 5E) as demonstrated by qRT-PCR assay. The immunohistochemical staining and western blotting analysis for EMT markers indicated that propofol diminished the level of N-cadherin and increased the level of E-cadherin in tumor tissue (Figure 5F, 5G). To evaluate the effect of propofol on cervical carcinoma cell metastasis *in vivo*, SiHa cells were injected into nude mice via tail vein injection. As shown in Figure 5H, 5I, propofol significantly inhibited lung metastatic ability of SiHa cells *in vivo*. Therefore, propofol suppressed tumor growth and metastasis of cervical carcinoma cell *in vivo*.

## DISCUSSION

Currently, propofol (2,6-diisopropylphenol) is one of the most commonly used anaesthetics. Intriguingly, mounting studies have disclosed that propofol has many non-anaesthetic effects, including anti-cancer activities [10]. Propofol decreases the level of circRNA transcriptional adaptor 2A (circTADA2A) and exerts anti-tumor effects in lung cancer [18]. Propofol epigenetically regulates trastuzumab resistance in breast carcinoma through intervening interleukin-6/miR-149-5p axis [19]. For gynecologic ovarian cancer, propofol-induced miR-125a-5p inhibits the growth and metastasis of ovarian carcinoma by declining Lin-28 Homolog B (LIN28B) [20]. In cervical cancer, propofol heightens the cisplatin-triggered cancer cell apoptosis via blocking EGFR/JAK2/STAT3 axis [21]. These studies have provided important piece of information on the anti-cancer activities of propofol, indicating that propofol may also be effective cancer therapeutic drug. Despite previous studies, however, the anti-metastatic mechanism of propofol in cervical cancer remains unclear.

Firstly, we evaluated the suppressive effect of propofol on the growth of cervical carcinoma cells. According to the results *in vitro*, we proved that propofol (30~80 μM) exerted significantly suppressive impact on the cell viability of cervical cancer cell lines (Caski and SiHa) in both a time-dependent pattern and a dose dependent manner. Consistently, propofol treatment caused more reductive effects in the colony formation of cervical cancer cells. Several events, including cell invasion, intravasation, extravasation, and metastasis formation are crucial for cancer cells to leave the primary tumor site and establish distant metastases [22, 23]. Therefore, transwell invasion assay was used to measure the effects of propofol on the invasion of cervical cancer cells *in vitro*. The results verified that propofol blocked the invasion of cervical cancer cells *in vitro* in a dose dependent manner.

Increasing evidence have corroborated that lncRNAs exert suppressive or promotion effects to modulate a variety of biological processes of various types of tumors [24]. To gain further insight into the potential mechanism of propofol in anticancer activity, we used dataset GSE6791 and GSE63514 from GEO database to screen the deregulated genes in primary cervical cancer tissue and normal tissue. To explore the biological activity of MIR155HG, we constructed MIR155HG silencing cervical cancer cell. The functional experimental results showed that knockdown of MIR155HG reduced the proliferation and invasive ability of cervical cancer cells. The transcription of MIR155HG is regulated by multiple transcription factors, such as v-myb myeloblastosis viral oncogene homolog (MYB), nuclear factor-ĸB (NF-κB) and activator protein-1 (AP-1) [25–27]. Several tumor types including cervical cancer show a persistent aberrant activation of NF-κB, and NF-κB activation promotes cancer invasion, metastasis, and chemoresistance [28]. MYB is overexpressed in several malignant tumors, including breast cancer, lung cancer and hepatocellular carcinoma, and is associated with tumor development [29]. The aberrantly expressed and constitutively active transcription factor AP-1 increases the severity of lesions during cervical carcinogenesis [30]. All these results suggest that the abnormally high expression of MIR155HG is closely related to these transcription factors. Next, we will focus on whether these transcription factors are participated in the anti-cancer effect of propofol.

Recent investigations have suggested that EMT is intently a key process during cancer cells invasion and metastasis [31]. EMT is a common pathological characteristic of colon, gastric and lung cancer, as well as other malignancies [32]. EMT alters the morphology of cancer cell, which exhibit pleomorphism and loss of cell polarity; such morphological features contribute to cell migration and invasion [33]. EMT is characterized by the down-regulation of E-cadherin, an epithelial marker; the up-regulation of mesenchymal markers, such as N-cadherin. Numerous studies have shown that lncRNA dysregulation plays key roles in human diseases, including cancer, by modulating the epithelial-mesenchymal transition (EMT) [34]. Few lncRNAs are already implicated as biomarkers and some of them are in clinical trials. they are potential targets for cancer therapy and there are several ways by which lncRNAs may be targeted to modulate their expression, including antisense oligonucleotide (ASO) [35]. Herein, we also found that knockdown of MIR155HG lessened the expression of N-cadherin and raised the expression E-cadherin in cervical cancer cells, indicating that knockdown of MIR155HG effectively suppressed EMT progression in cervical carcinoma cells. Intriguingly, after cervical cancer cells were treated with propofol, we validated that MIR155HG level was significantly decreased.

To further explore the anti-tumor effectiveness of propofol *in vivo*, SiHa xenograft model was applied. Compared with the vehicle group, without altering the body weight of mice, propofol decreased both tumor volume and tumor weight. Subsequently, by qRT-PCR, the level of MIR155HG in tumor tissue from mice treated with propofol was decreased, indicating that MIR155HG may be involved in the anti-tumor effect of propofol. Consistent with the results *in vitro*, the expression of E-cadherin was increased, and N-cadherin in tumor tissue was decreased after propofol treatment, which indicated that propofol blocked the EMT process of cervical cancer cells *in vivo*. Moreover, upregulation of MIR155HG via pCDNA3.1-MIR155HG plasmid abrogated the effect of propofol in cervical cancer cells, suggesting MIR155HG was the potential target for propofol. When cells overexpressing MIR155HG were treated with propofol, the inhibitory impacts of propofol on the growth and invasion abilities of cervical cancer cells were diminished, which confirmed that propofol suppressed aggressive traits of cervical cancer cells by reducing MIR155HG. Collectively, our study demonstrated that propofol blocks the growth and invasive traits of cervical carcinoma cell via inhibiting MIR155HG.
